# Diversity in chemosensory receptor genes in dogs and wolves: degeneration of the olfactory receptor gene repertoire in the brachycephalic Pug

**DOI:** 10.1093/chemse/bjaf062

**Published:** 2025-12-15

**Authors:** Hyuga Inoue, Matthew Gibbs, Scott J McGrane, Yoshihito Niimura

**Affiliations:** Department of Veterinary Sciences, Faculty of Agriculture, University of Miyazaki, 1-1, Gakuen Kibanadai Nishi, Miyazaki, Miyazaki 889-2192, Japan; Waltham Petcare Science Institute, Mars Petcare, Waltham on the Wolds, Melton Mowbray, Leicestershire, LE14 4RT, United Kingdom; Waltham Petcare Science Institute, Mars Petcare, Waltham on the Wolds, Melton Mowbray, Leicestershire, LE14 4RT, United Kingdom; Department of Veterinary Sciences, Faculty of Agriculture, University of Miyazaki, 1-1, Gakuen Kibanadai Nishi, Miyazaki, Miyazaki 889-2192, Japan

**Keywords:** single nucleotide variant, copy number variation, gene family evolution, domestication, vomeronasal receptor, taste receptor

## Abstract

Dogs, domesticated from gray wolves over 15,000 yr ago, exhibit extensive variation among breeds, including differences in olfactory ability. To investigate the genetic basis of these differences, we analyzed single-nucleotide variants (SNVs) in 3 chemosensory receptor gene families—olfactory receptors (ORs), vomeronasal receptors type 1 (V1Rs), and bitter taste receptors (T2Rs)—using whole-genome data from the Dog Biomedical Variant Database Consortium, which includes 635 domestic dogs representing 121 breeds and 8 wolves. We identified 179 segregating pseudogenes in OR genes (minor allele frequency > 1%), including cases where intact genes are pseudogenized in some individuals and vice versa. The number of functional OR genes varied substantially among individuals (779 to 807), while V1R and T2R gene counts were nearly invariant (8 and 16, respectively). Compared to wolves, dogs exhibited significantly higher ratios of nonsynonymous to synonymous SNVs (N/S) in OR and T2R genes, suggesting relaxed functional constraints potentially associated with domestication. Among breeds, Pugs had significantly fewer functional OR genes and a higher N/S ratio than other breeds, even after accounting for copy number variation. Notably, an OR gene orthologous to the human androstenone receptor, OR7D4, was completely pseudogenized in all Pugs but remained largely functional in other breeds. These findings support the hypothesis that reduced olfactory ability in brachycephalic breeds, such as Pugs, is associated with genetic degeneration of OR genes. Overall, our study provides new insights into the genetic diversity of chemosensory receptor repertoires in dogs and underscores the impact of domestication and breed-specific morphological traits on olfactory function.

## 1. Introduction

Dogs (*Canis lupus familiaris*) are believed to have been domesticated from gray wolves (*Canis lupus*) in Eurasia at least 15,000 yr ago, although the exact timing, location, and process of domestication remain subjects of ongoing debate (Bergström et al. 2022; Gojobori et al. 2024). Today, there are nearly 400 dog breeds, each characterized by distinct traits related to morphology (e.g. body size and skull shape), behavior, and even characters. Among them, “modern” breeds were developed over the past few 100 yr by selective breeding (Ostrander et al. 2017). Generally, each breed forms a genetically distinct clade within the dog phylogeny (vonHoldt et al. 2010; Parker et al. 2017; Morrill et al. 2022). These breeds are classified into several groups—such as scent hounds, sight hounds, and retrievers—based on both primary purposes and genealogical relationships.

Dogs are well known for their remarkable sense of smell. Scent hounds, in particular, have been selectively bred for olfactory performance and include breeds, such as Beagles, Basset Hounds, and Bloodhounds. These breeds, along with others like Labrador Retrievers and German Shepherds, are widely trained and employed for detecting drugs (Jezierski et al. 2014), explosives (Gazit et al. 2003), missing persons during disasters (Fenton 1992), and individuals with medical conditions, such as cancer (Pirrone and Albertini 2017) and COVID-19 (Grandjean et al. 2020; Jendrny et al. 2020).

In contrast, some dog breeds have been selected primarily for their distinctive and desirable appearance. For example, brachycephalic breeds such as Pugs and Bulldogs were bred for their exaggerated short-nosed facial features. Brachycephalic dogs are generally assumed to have impaired olfactory function due to the shortened rostrum, which likely reduces airflow to the nasal cavity (Packer et al. 2015). Additionally, brachycephaly has been associated with a repositioning of the olfactory lobe, which may negatively affect olfactory capabilities (Roberts et al. 2010).

Several studies have examined differences in olfactory ability across dog breeds and between dogs and wolves. Polgár et al. (Polgár et al. 2016) developed training-free olfactory tests to assess olfactory capacity in 4 groups: (i) scent hounds, (ii) brachycephalic dogs, (iii) other dog breeds, and (iv) hand-reared gray wolves. They found that groups (i) and (iv) outperformed groups (ii) and (iii). In contrast, Hall et al. (2015) reported different results: they compared the olfactory ability of German Shepherds, Pugs, and Greyhounds (sight hounds), finding that Pugs significantly outperformed German Shepherds in odor discrimination tasks, especially under diluted odorant concentrations, while no significant differences were observed in vision-based tasks. They suggested that, contrary to general assumptions, brachycephalic dogs may have superior olfactory performance, although behavioral motivation rather than olfactory capacity may have contributed to these results.

The mammalian olfactory system comprises 2 subsystems: the main olfactory system (MOS) and the accessory olfactory system (AOS), also known as the vomeronasal system. In the MOS, olfactory receptor (OR) genes are expressed in the main olfactory epithelium (MOE) of the nasal cavity, where they detect volatile odorants (Buck and Axel 1991; Niimura et al. 2020). OR genes constitute the largest multigene family in mammals, with the numbers of genes varying widely across species. For example, humans, mice, and African elephants possess approximately 400, 1,100, and 2,000 functional OR genes, respectively (Niimura and Nei 2003; Niimura et al. 2014, 2018; Policarpo et al. 2024). Dogs, interestingly, have an intermediate number, with about 810 functional OR genes.

Comparative analyses of OR gene repertoires across mammalian species have revealed extensive lineage-specific gene gains and losses throughout evolution (Niimura and Nei 2007; Hayden et al. 2010; Niimura et al. 2014, 2018; Hughes et al. 2018). The estimated rates of OR gene birth and death are substantially higher than the average among all mammalian gene families (Niimura et al. 2014). The OR gene family represents a striking example of birth-and-death evolution, in which new genes arise through duplication, some of which are retained over long evolutionary timescales, while others are lost or rendered nonfunctional due to deleterious mutations (Nei and Rooney 2005; Nei et al. 2008).

In humans, OR gene coding regions are highly polymorphic (Olender et al. 2012; Mainland et al. 2014) and exhibit considerable copy number variation (CNV) among individuals (Nozawa et al. 2007; Young et al. 2008; Shadravan 2013; Akhtar et al. 2022). Olender et al. (2012) analyzed single-nucleotide variant (SNV) data from the 1000 Genomes Project and identified 244 segregating OR pseudogenes, in which both intact and pseudogene alleles coexist at the same locus. Mainland et al. (2014) investigated nonsynonymous SNVs at intact OR loci and estimated that, on average, any 2 individuals differ functionally at more than 30% of their OR gene alleles. Additionally, OR gene expression patterns vary significantly between individuals. Verbeurgt et al. (2014) examined the expression of 356 intact OR genes in whole olfactory mucosa tissues from 26 individuals. On average, 273 OR genes were expressed per individual, but only 90 genes were expressed in all individuals. Collectively, these studies demonstrate that the repertoire of functional OR genes varies remarkably among human individuals, both at the genomic and transcriptomic levels.

In the AOS of rodents, 2 types of vomeronasal receptor genes—V1Rs and V2Rs—are expressed in spatially distinct layers of the vomeronasal organ (VNO): V1Rs in the apical layer and V2Rs in the basal layer. V1Rs are generally tuned to small molecules, including volatile compounds and steroid-derived ligands, whereas V2Rs respond to larger ligands such as peptides and proteins (Tirindelli 2021; Murata et al. 2024). The numbers of V1R and V2R genes vary extensively among mammalian species (Young and Trask 2007; Young et al. 2010). In dogs, only about 8 to 9 functional V1R genes have been reported (Grus et al. 2005; Young et al. 2010). Notably, in many mammalian species including dogs, the V2R gene family is entirely pseudogenized (Young and Trask 2007; Policarpo et al. 2024). Furthermore, immunohistochemical studies in the dog VNO failed to detect expression of Gαo—the G-protein typically coupled to V2Rs, suggesting that the canine VNO may rely exclusively on V1R-mediated signal transduction (Salazar et al. 2013; Barrios et al. 2014).

Olfaction and taste are complementary chemical senses. There are 5 basic taste modalities: sweet, umami, bitter, salty, and sour. Among them, sweet, umami, and bitter are mediated by taste receptors type 1 (T1Rs) and type 2 (T2Rs), which are expressed in the taste buds of the tongue (Yarmolinsky et al. 2009). T1Rs form heterodimers to detect sweet and umami stimuli, while T2Rs function as monomers that recognize bitter substances. While the number of T2R genes varies widely among species (Li and Zhang 2014; Shang et al. 2017), the number of T1R genes is generally conserved at 3 copies in most mammals, including dogs, with some exceptions (Wolsan and Sato 2022; Policarpo et al. 2024).

All of these receptor genes—OR, V1R, and T2R—belong to the G protein-coupled receptor superfamily and share a common 7-transmembrane structure. OR, V1R, and T2R genes also exhibit similar structural characteristics: they are approximately 1,000 base pairs in length and typically lack introns in their coding regions. Interestingly, V1R and T2R genes share weak sequence homology. A recent study (Niimura et al. 2024) demonstrated that V1R genes have undergone the most rapid evolution, followed by OR genes, whereas T2R genes have remained the most evolutionarily conserved. This variation in evolutionary rate likely reflects differences in the nature of their ligands: species-specific pheromones for V1Rs, environment-dependent scents for ORs, and broadly conserved toxic substances for T2Rs.

Although several studies have investigated chemosensory receptor gene variation among dog breeds (Tacher et al. 2005; Lesniak et al. 2008; Robin et al. 2009; Yang et al. 2016, 2022), these studies have typically analyzed only a limited number of OR genes: 16 in Tacher et al. (2005), 5 in Lesniak et al. (2008), 109 in Robin et al. (2009), 12 in Yang et al. (2016), and 7 in Yang et al. (2022). More recently, Mouton et al. (2025) analyzed CNVs in OR genes as well as olfactory anatomy across 111 domestic dogs from 30 breeds, 27 gray wolves, and 4 coyotes. Their study showed that wolves and coyotes possessed significantly larger OR gene repertoires than domestic dogs but detected no significant differences in OR gene numbers among dog breeds, except for dingoes. In particular, they found no evidence of superiority of scent hounds over other breeds in either the size of the OR gene repertoire or the relative size of the cribriform plate (CP). These findings suggest that the superior olfactory performance of scent-detection dogs likely reflects behavioral traits such as motivation and trainability rather than genomic or anatomical differences. Regarding V1R and T2R genes, no comprehensive studies have yet investigated variation in these genes among dog breeds or between dogs and wolves.

In this study, we aimed to examine the variation in chemosensory receptor genes across dog breeds and between dogs and wolves, in order to explore a potential association with olfactory ability. We analyzed the repertoires of OR, V1R, and T2R genes using SNV data from 635 domestic dogs representing 121 breeds, 8 gray wolves, and 16 canine genome assemblies. Our results revealed that the brachycephalic Pug possess a degenerated OR gene repertoire compared to other breeds analyzed.

## 2. Materials and methods

### 2.1 Data

The Dog Biomedical Variant Database Consortium (DBVDC) database (Jagannathan et al. 2019) was used to analyze SNVs. All contributors to the DBVDC gave consent for the inclusion of their data in the database and its use for research. Mars Petcare UK is a contributor of dog genomes to the database. Chemosensory receptor data was extracted for the purpose of this research. At the time of this analysis, this database contained SNV data for 640 dog individuals from 121 breeds, as well as 8 wolf individuals, all mapped to the CanFam3.1 dog genome assembly (Supplementary Table S1). Among the 640 dog individuals, 5 were excluded from the analysis because more than 50% of SNVs in OR genes were missing for these individuals. As a result, 635 dog individuals were included in this study.

In addition, we downloaded 16 dog/wolf genome assemblies from GenBank (https://www.ncbi.nlm.nih.gov/genbank/), which were assembled at the chromosome level with a contig N50 greater than 1 Mb (excluding CanFam3.1). These genome assemblies included: Boxer (CanFam3.1 and Dog10K_Boxer_Tasha), Labrador Retriever (ROS_Cfam_1.0 and Yella_v2), German Shepherd (ASM864105v3 and UU_Cfam_GSD_1.0), Basenji (UNSW_CanFamBas_1.2 and Basenji_breed-1.1), Dingo (ASM325472v2 and UNSW_AlpineDingo_1.0), Bernese Mountain Dog (BD_1.0 and OD_1.0), Cairn Terrier (CA611_1.0), Great Dane (UMICH_Zoey_3.1), and Gray Wolf (mCanLor1.2 and Clu-1) (Supplementary Table S2).

### 2.2 Identification of OR, V1R, and T2R genes

OR genes were identified from the genome assemblies using the method previously reported by Niimura (2013), with modifications as in Niimura et al. (2018, 2024). V1R and T2R genes were identified following the procedure of Niimura et al. (2024).

Briefly, we first performed TBLASTN searches (Altschul et al. 1997) against the whole-genome sequences using representative gene sequences as queries, with an e-value cutoff of 1e-20. For the identification of OR genes, 781 consensus sequences of placental mammals were used as queries, whereas 318 V1R, 8 ancV1R, and 158 T2R genes from various mammalian species were used for identifying V1R and T2R genes. Each BLAST hit was then extended along the genomic DNA to locate an initiation codon at an appropriate position and a stop codon downstream. A sequence containing an internal stop codon, frameshift, and/or deletion in a conserved region (e.g. the 7 transmembrane regions) was regarded as a pseudogene, whereas a sequence having an intact coding region that begins with an initiation codon and ends with a stop codon was regarded as a functional gene. Partial intact sequences were counted separately as truncated genes because their functionality could not be determined at this stage.

The genomic coordinates of the identified genes are provided in Supplementary Table S3.

### 2.3 Analysis of SNVs

SNVs located within the coding regions of OR, V1R, and T2R genes (including pseudogenes) were extracted from the DBVDC database. Of the 816 functional OR genes identified, 3 were located on the X chromosome and were excluded from the analyses because X-linked loci may evolve differently from autosomal loci (the faster-X effect) (Meisel and Connallon 2013) and because our dataset included both males and females with some individuals of unknown sex. No functional V1R or T2R genes were X-linked. In addition, 7 OR genes located on autosomes or unplaced short contigs lacked SNV information in the database and were therefore excluded. In total, SNVs in functional genes of 806 ORs, 8 V1Rs, and 16 T2Rs were analyzed. We also examined 32 OR, 1 V1R, and 1 T2R pseudogenes with a single premature stop codon or frameshift to assess whether they retain potentially functional alleles.

For each gene, nucleotide sequences were reconstructed by incorporating identified SNVs and subsequently translated into amino acid sequences. Based on these translations, SNVs that resulted in pseudogenization were identified, including frameshift mutations, nonsense mutations introducing premature stop codons, and mutations affecting the start codon. All other SNVs were categorized as nonsynonymous substitutions, synonymous substitutions, or insertion/deletions that do not cause frameshifts.

In cases where 2 pseudogenizing SNVs were present within the same gene in a single individual, we assumed both mutations occurred on the same allele, rather than being distributed across the 2 alleles (Supplementary Fig. S1). This assumption reflects the higher likelihood that, once a gene has been pseudogenized, additional pseudogenizing mutations may accumulate more readily on the same allele.

For analyses presented in Figs. 3 and 4, the mean number of synonymous or nonsynonymous SNVs across the 2 alleles was calculated for each individual, such that a heterozygous SNV was counted as 0.5.

### 2.4 Identification of CNV groups

CNV groups for OR genes were identified as follows. During OR gene identification, we employed 781 query sequences for homology searches. Each query sequence represented a consensus sequence of an orthologous gene group (OGG) among placental mammals (Niimura et al. 2014, 2018). Accordingly, for each OR gene identified in dogs and wolves in this study, the closest (“best-hit”) OR gene query was assigned. We then classified the identified dog/wolf OR genes from the 15 genome assemblies (excluding Dog10K_Boxer_Tasha) into OGGs based on their best-hit ORs.

Next, we constructed a phylogenetic tree for each OGG separately. From each tree, we extracted the smallest clades that were supported by bootstrap values greater than 98% and contained genes from all 15 genome assemblies; these clades were defined as CNV groups. Genes showing less than 98% sequence identity were classified into separate groups. All phylogenetic trees were visually inspected to confirm the CNV groups (Supplementary Fig. S2). As a result, we identified 825 CNV groups for OR genes (Supplementary Table S4).

For V1R and T2R genes, phylogenetic trees were constructed using all genes identified from the 15 dog/wolf genome assemblies. Clades supported by bootstrap values greater than 98% and containing genes from multiple genome assemblies were extracted (Supplementary Fig. S3). Consequently, we identified 7 and 18 CNV groups for V1R and T2R genes, respectively (Supplementary Tables S5 and S6).

### 2.5 Construction of phylogenetic trees

Neighbor-joining phylogenetic trees (Saitou and Nei 1987) were constructed from translated amino acid sequences using the LINTREE program (Takezaki et al. 1995) with Poisson correction distance. Multiple sequence alignments of amino acid sequences were performed using MAFFT (Katoh and Standley 2013).

### 2.6 Statistical analysis

We categorized 121 dog breeds into 2 ways: (i) 21 breed groups according to the classification of the Fédération Cynologique Internationale (FCI; https://www.fci.be/en/) and (ii) 3 skull-shape types based on the cephalic index—brachycephalic, mesocephalic, and dolichocephalic breeds—according to O'Neill et al. (2020).

The numbers of functional OR gene loci, unique functional OR genes, and N/S ratios were compared among different breeds or categories using 1-way analysis of variance (ANOVA). Because some breeds or categories contained a small number of samples (Supplementary Table S1), we determined the minimum sample size required for each category according to Table 8.4.6 in (Cohen 1988). To detect a medium effect (*f* = 0.25) with α = 0.05 and power = 0.80, the required sample sizes per category are 22 and 20 for *u* = 12 and *u* = 15, respectively, where *f* denotes Cohen's *f* (effect size) and *u* represents the degree of freedom (number of categories − 1). To detect a large effect (*f* = 0.4) under the same conditions, the required sample sizes are 9 and 8 for *u* = 12 and *u* = 15, respectively. Therefore, we included only categories with *n* ≥ 10 in the ANOVA and subsequent statistical analyses. Among these, Tukey's honest significant difference (HSD) test was used for multiple comparisons.

## 3. Results

### 3.1 OR, V1R, and T2R genes in dog genomes

We identified 816, 8, and 16 functional OR, V1R, and T2R genes, respectively, from the CanFam3.1 dog genome assembly (Table 1). These numbers are largely consistent with previous reports (Niimura and Nei 2007; Young et al. 2010; Shang et al. 2017; Gibbs et al. 2022). Of the 816 OR genes identified, 10 were excluded from the analysis (3 located on the X chromosome and 7 lacking SNP data; see Materials and Methods). In total, SNVs located within the coding regions of 806 OR, 8 V1R, and 16 T2R genes retrieved from the DBVDC database were analyzed for 635 dogs and 8 wolves.

**Table 1.** Numbers of OR, V1R, and T2R genes in the 16 genome assemblies.

Genome	Breed	OR				V1R			T2R		
		F	T	P	U	F	P	U	F	P	U
**CanFam3.1**	Boxer	816	0	276	781	8	28	7	16	4	16
**Dog10K_Boxer_Tasha**	Boxer	684	0	251	—	6	27	—	17	3	—
**ROS_Cfam_1.0**	Labrador retriever	803	1	343	772	6	28	6	17	3	17
**Yella_v2**	Labrador retriever	794	1	255	779	8	28	7	17	3	17
**ASM864105v3**	German Shepherd	811	0	282	789	7	27	7	17	4	17
**UU_Cfam_GSD_1.0**	German Shepherd	817	2	368	759	8	27	7	17	3	17
**BD_1.0**	Bernese Mountain Dog	730	0	293	722	7	28	7	16	4	16
**OD_1.0**	Bernese Mountain Dog	772	1	261	763	7	28	7	16	6	16
**CA611_1.0**	Cairn Terrier	801	0	246	783	7	28	7	17	3	17
**UMICH_Zoey_3.1**	Great Dane	696	3	392	683	8	28	7	16	3	16
**UNSW_CanFamBas_1.2**	Basenji	790	0	266	777	6	28	6	18	2	18
**Basenji_breed-1.1**	Basenji	759	0	384	715	6	30	6	18	2	18
**ASM325472v2**	Dingo	771	0	275	748	8	27	7	17	3	17
**UNSW_AlpineDingo_1.0**	Dingo	761	0	365	730	8	28	7	15	5	15
**mCanLor1.2**	Gray wolf	861	0	255	802	7	27	7	17	3	17
**Clu-1**	Gray wolf	760	0	305	738	8	27	7	17	3	17

F, T, P, and U indicate the number of functional genes, truncated genes, pseudogenes, and unique genes, respectively.

Numbers of unique genes for Dog10K_Boxer_Tasha were not determined.

The mean numbers of SNVs per an OR gene, a V1R gene, and a T2R gene were 18.9, 21.8, and 10.0, respectively (including all SNVs present in at least one allele). Since the lengths of these genes are nearly the same (∼1 kb), OR and V1R genes appear to be more polymorphic than T2R genes.

**Fig. 1.**
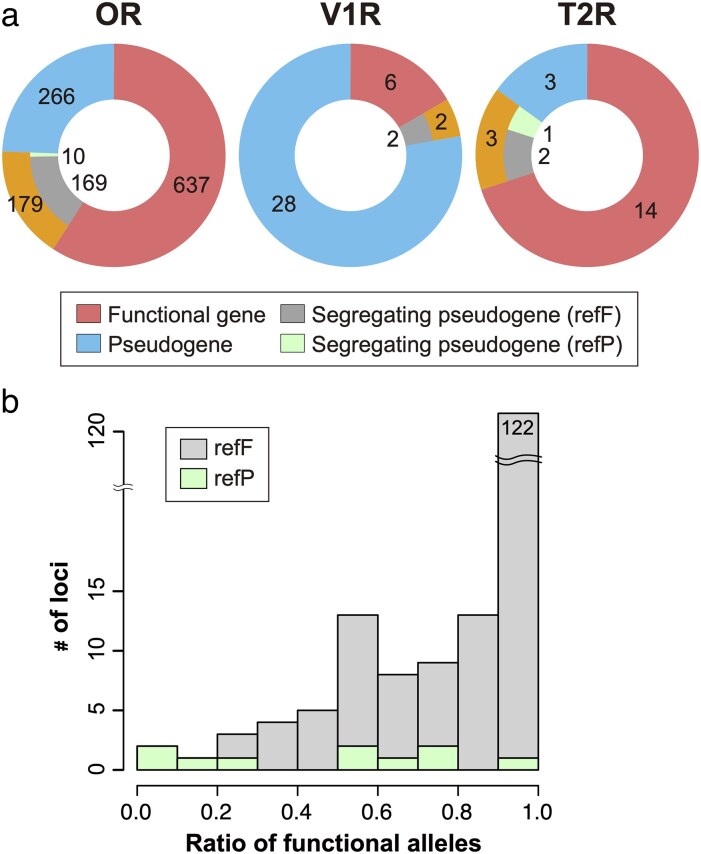
Segregating pseudogenes in dogs. (a) Numbers of functional genes, segregating pseudogenes (with MAF > 1%), and pseudogenes for OR, V1R, and T2R genes. Segregating pseudogenes were further classified into 2 categories: refF, functional in the CanFam3.1 assembly; and refP, pseudogenized in the CanFam3.1 assembly. (b) Histogram showing the proportion of functional alleles among the 1,286 alleles from 635 dogs and 8 wolves for the 179 OR segregating pseudogenes. The bars for refF and refP genes are shown separately.

A segregating pseudogene is defined as a gene that remains functional in some alleles but is pseudogenized in others within the population. Among the 806 OR genes functional in the CanFam3.1 assembly, 169 were identified as segregating pseudogenes with a minor allele frequency (MAF) greater than 1% in the DBVDC data (Fig. 1a). Additionally, 10 out of 276 OR pseudogenes in CanFam3.1 were also segregating pseudogenes with MAF > 1%. In total, we identified 179 segregating pseudogenes, the majority of which are intact in CanFam3.1 (Fig. 1a). For V1R and T2R genes, 2 and 3 segregating pseudogenes with MAF > 1% were identified, respectively. Figure 1b shows the proportion of functional alleles among 1,286 alleles in 643 individuals (635 dogs and 8 wolves) for the 179 OR segregating pseudogenes. Most segregating pseudogenes remain functional in the majority of alleles: 122 out of 179 are functional in more than 90% of alleles.

Note that each individual carries 2 alleles at a given gene locus. If at least one allele encodes a functional gene, the gene locus is considered functional in that individual (see Supplementary Fig. S1). Only when both alleles are pseudogenized is the gene locus considered non-functional. Using this approach, we counted the number of functional loci for OR, V1R, and T2R genes for each individual.

**Fig. 2.**
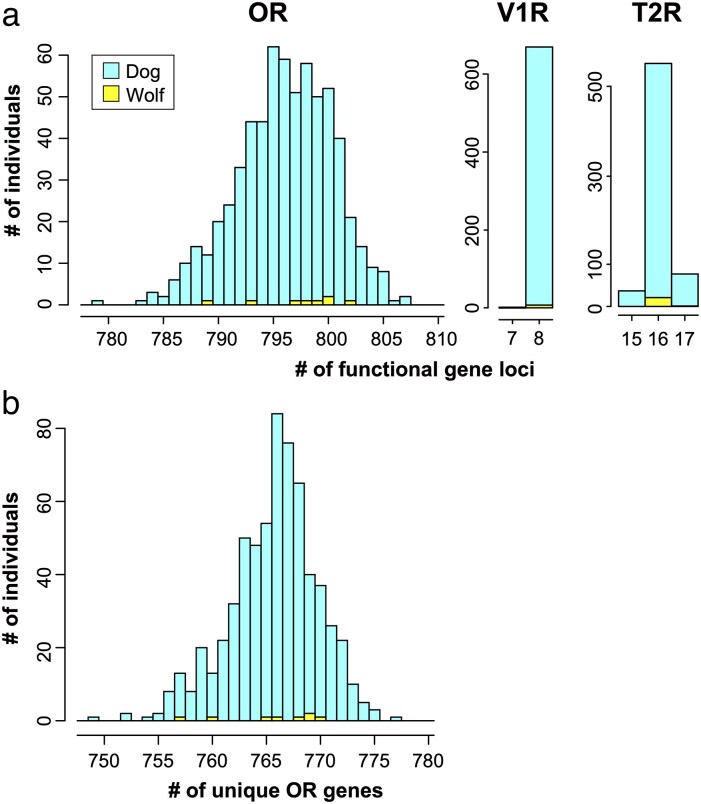
Distribution of the number of functional genes across the 635 dog and 8 wolf individuals. (a) Number of functional gene loci for OR, V1R, and T2R genes. Bars for dog and wolf genes are shown separately. (b) Number of unique OR genes.

Figure 2a shows the distribution of the number of functional OR gene loci among the 643 individuals analyzed (635 dogs and 8 wolves). The number of functional OR loci ranged from 779 to 807, with a standard deviation (SD) of 4.34. In contrast, the numbers of functional V1R and T2R loci were more consistent: 99.7% and 83.0% of individuals possessed 8 and 16 functional loci, respectively. For V1R, only one dog and one wolf carried 7 loci, while all others had 8. The SDs for the number of functional V1R and T2R loci were 0.056 and 0.41, respectively.

Because the DBVDC database is based on mapping SNVs to the CanFam3.1 reference genome, it cannot capture CNVs involving duplicated genes with nearly identical sequences. Furthermore, if an individual possesses an OR gene absent from CanFam3.1, its SNVs may be incorrectly assigned to the most similar OR gene present in CanFam3.1. Therefore, to evaluate CNVs, we analyzed 13 additional dog genome assemblies and 2 wolf assemblies independently assembled from CanFam3.1. OR, V1R, and T2R genes were identified and categorized as intact, truncated, or pseudogenes (Niimura and Nei 2007). As a result, we identified 684 to 861 intact OR genes, 6 to 8 V1R genes, and 15 to 18 T2R genes across these assemblies (Table 1).

Notably, the Dog10K_Boxer_Tasha assembly—the updated version of CanFam3.1—harbors substantially fewer functional OR genes (684) compared to CanFam3.1 (816). Detailed comparison revealed that a large number of OR genes present in CanFam3.1 are absent from Dog10K_Boxer_Tasha (Supplementary Fig. S4). Therefore, we did not use the Dog10K_Boxer_Tasha assembly for further analyses.

### 3.2 Number of unique genes

We next counted “unique” OR, V1R, and T2R genes to account for putative CNVs. If an individual genome contains multiple highly similar gene copies, the presence of such redundant copies may not directly contribute to the individual's chemosensory ability. Therefore, we defined a “CNV group” as a set of highly similar genes and used the number of CNV groups as a proxy for the number of unique genes. Specifically, a CNV group was defined as a set of genes that are more similar to each other than the expected sequence identity between different alleles at the same locus, as determined by phylogenetic relationships and sequence identity analyses (see Materials and Methods). The number of unique genes is expected to better reflect the chemosensory repertoire of each individual than the total number of functional genes present in the genome.

As a result, we classified 11,742 OR, 109 V1R, and 251 T2R genes from the 15 genome assemblies (including CanFam3.1 but excluding Dog10K_Boxer_Tasha) into 825, 7, and 18 CNV groups, respectively (Supplementary Tables S4–S6). We then counted the number of unique genes for OR, V1R, and T2R genes in each genome assembly. In the CanFam3.1 assembly, 781 OR, 7 V1R, and 16 T2R unique genes were identified (Table 1); thus, 44 OR, 0 V1R, and 2 T2R CNV groups are absent from the CanFam3.1 assembly. Across the 15 genome assemblies, the number of unique genes ranged from 683 to 802 for OR genes, 6 to 7 for V1R genes, and 15 to 18 for T2R genes (Table 1). A wolf genome assembly, mCanLor1.2, contained the largest number of functional OR genes (861) and unique OR genes (802) among the 15 genomes examined. Detailed comparative analyses identified several gene duplications within CNV groups on chromosomes 6, 9, and 27 (Supplementary Fig. S5). For V1R genes, only one CNV group contained multiple (2) V1R genes (Supplementary Fig. S3, left). For T2R genes, the number of unique genes was identical to the number of functional genes in all examined genomes; thus, all 18 T2R genes identified in this study are unique (Supplementary Fig. S3, right).

We also analyzed the number of unique genes in each individual in the DBVDC database (Fig. 2b). The number of unique OR genes ranged from 749 to 777, with a SD of 4.04, which is slightly smaller than the SD (4.34) observed for the number of functional OR gene loci.

### 3.3 Comparison between wolves and dogs

**Fig. 3.**
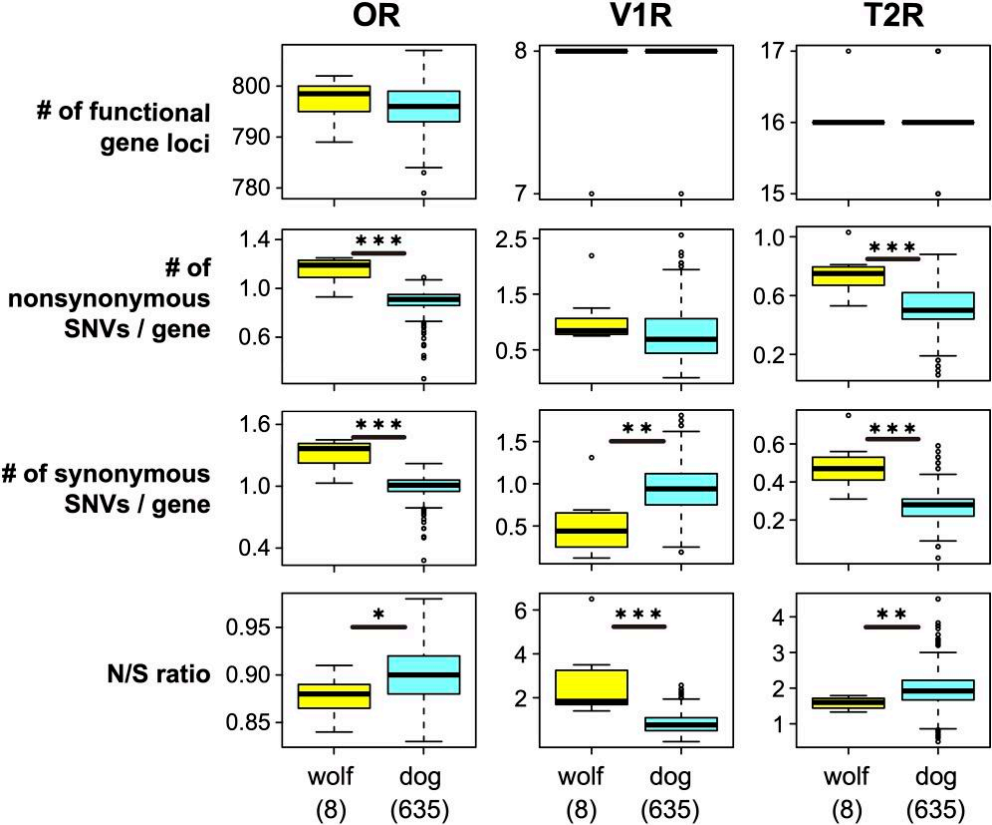
Comparison of the numbers of functional gene loci, nonsynonymous SNVs per gene, synonymous SNVs per gene, and N/S ratios for OR, V1R, and T2R genes. Blue and yellow boxes represent data for 635 dogs and 8 wolves, respectively. ***P < 0.001; **P < 0.01; *P < 0.05; Wilcoxon rank-sum test.

To investigate genetic changes in OR, V1R, and T2R genes associated with domestication, we compared gene repertoires between wolves and dogs. No significant differences were observed in the numbers of functional gene loci for OR, V1R, and T2R genes between wolves and dogs (Fig. 3, top; all *P*-values > 0.05, Wilcoxon rank-sum test). Similarly, there were no significant differences in the number of unique OR genes (Supplementary Fig. S6; *P* = 0.80, Wilcoxon rank-sum test).

For SNV analysis, the number of synonymous and nonsynonymous SNVs at each locus in an individual was calculated as the mean of the 2 alleles at that locus (see Materials and Methods). As expected, for OR and T2R genes, both the numbers of synonymous and nonsynonymous SNVs were significantly lower in dogs than in wolves (Fig. 3, second and third panels). This difference likely reflects the fact that the reference genome (CanFam3.1) was derived from a Boxer dog; because domestic dogs originated from a subgroup of gray wolves, wolf genomes are generally more divergent from the reference than dog genomes.

However, when we examined the ratio of nonsynonymous to synonymous SNVs (N/S), an opposite pattern emerged: N/S ratios were higher in dogs than in wolves for both OR and T2R genes. This suggests that functional constraints on these genes may have been relaxed in dogs, possibly as a result of domestication (see Discussion).

In contrast, the N/S ratio for V1R genes showed the opposite tendency compared to OR and T2R genes. The reason for this observation remains unclear, but may reflect the smaller number of genes for V1R (8) compared to OR (806) or T2R (16).

### 3.4 Comparison among dog breed groups

We conducted a comparative analysis of OR genes across 21 groups of dog breeds classified according to the FCI system (Supplementary Fig. S7; Supplementary Table S1). A 1-way ANOVA using 14 breed groups with ≥10 individuals revealed a significant difference among groups (*P* = 0.0001), and Tukey's HSD test identified 4 pairwise comparisons with significant differences (*P* < 0.05; Supplementary Table S7; see Materials and Methods).

Notably, scent hounds did not possess a significantly higher number of functional OR genes compared with other breeds (*P* = 0.99, Wilcoxon rank-sum test). Similarly, no significant difference was observed between scent hounds and sighthounds (*P* = 0.45, Wilcoxon rank-sum test).

For V1R and T2R genes, most groups exhibited identical gene counts, with 8 and 16 functional genes, respectively (Supplementary Table S7).

### 3.5 Comparison among skull-shape types

We also categorized dog breeds into 3 skull-shape types: brachycephalic (15 breeds, 56 individuals), mesocephalic (58 breeds, 368 individuals), and dolichocephalic (21 breeds, 95 individuals). ANOVA using individual values revealed significant differences among the 3 categories in the numbers of functional OR gene loci, unique functional OR genes, and N/S ratios (Supplementary Fig. S8, left). Tukey's HSD tests indicated that dolichocephalic breeds had significantly fewer functional OR gene loci than mesocephalic breeds. Both brachycephalic and dolichocephalic breeds also had significantly fewer unique functional OR genes and higher N/S ratios than mesocephalic breeds.

However, these differences were no longer significant when breed means were used instead of individual values (Supplementary Fig. S8, right). Because the number of individuals per breed was uneven, with 20 of the 56 brachycephalic individuals being Pugs, the significant difference for brachycephalic breeds likely reflects the strong contribution of Pugs.

### 3.6 Comparison among dog breeds

**Fig. 4.**
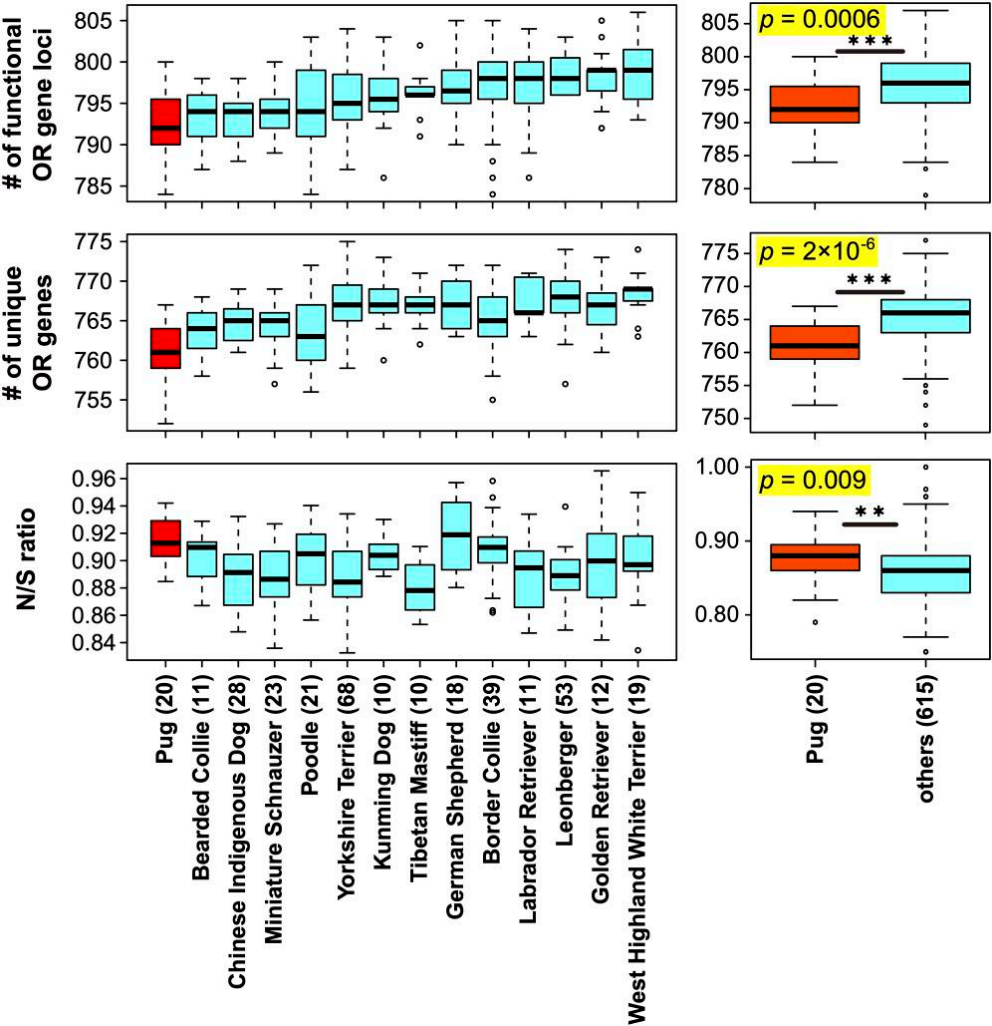
Comparison of the numbers of functional OR genes and N/S ratios among dog breeds (*n* ≥ 10, left) and between Pugs and the other breeds (right). Pug data are shown in red. ***P < 0.001; **P < 0.01; Wilcoxon rank-sum test.

Next, we examined breed-specific differences in the number of OR genes. Because some breeds had small sample sizes, we restricted the analysis to 14 breeds with at least 10 individuals (Supplementary Fig. S9; see Materials and Methods). ANOVA revealed a significant difference among the 14 breeds (*P* = 2 × 10^−8^; Fig. 4, top). Among them, Pugs—a brachycephalic breed—had the fewest functional OR gene loci. Tukey's HSD tests identified 5 breeds with significantly higher values than Pugs (Supplementary Table S7). When Pugs were compared with all other dog individuals, the difference remained significant (*P* = 0.0006, Wilcoxon rank-sum test; Fig. 4, top).

When comparing the numbers of unique functional OR genes, the significance became even stronger. ANOVA using the same 14 breeds showed a significant difference among them (Fig. 4, middle; *P* = 2 × 10^−15^). Tukey's HSD tests identified 10 breeds with significantly larger numbers than Pugs (Supplementary Table S7). Moreover, Pugs had significantly fewer unique functional OR genes than all other breeds (Fig. 4, middle; *P* = 2 × 10^−6^, Wilcoxon rank-sum test).

We also compared the N/S ratios of functional OR genes among dog breeds to assess potential relaxation of selective constraints. Since pseudogenized alleles typically exhibit higher N/S ratios than functional alleles, including them in the analysis could confound the results—elevated N/S ratios might simply reflect a greater number of pseudogenes rather than relaxed functional constraint. To avoid this bias, we restricted our analysis to 587 OR gene loci that were functional in all 643 individuals examined. ANOVA revealed a significant difference among the 14 breeds (Fig. 4, bottom; *P* = 5 × 10^−9^), and Tukey's HSD tests identified 5 breeds with significantly lower values than Pugs. Moreover, Pugs exhibited a significantly higher N/S ratio than other breeds (*P* = 0.009), suggesting that the selective pressure to maintain functional OR genes may be relaxed in Pugs.

To investigate whether pseudogenization in Pugs occurred at specific OR loci, we analyzed 20 Pug individuals and found that 54 loci were non-functional (i.e. both alleles pseudogenized) in at least one individual. We then performed a permutation test by randomly selecting 20 individuals from the full dataset of 635 dogs, counting the number of loci that were non-functional in at least one individual, and repeating this process 1,000 times (Supplementary Fig. S10). The resulting distribution was approximately normal, with a mean of 86.3 and an SD of 7.79. The observed number in 20 Pugs (54) was significantly lower than expected (*P* = 1 × 10^−5^), suggesting that the same OR genes tend to be pseudogenized in this breed.

**Fig. 5.**
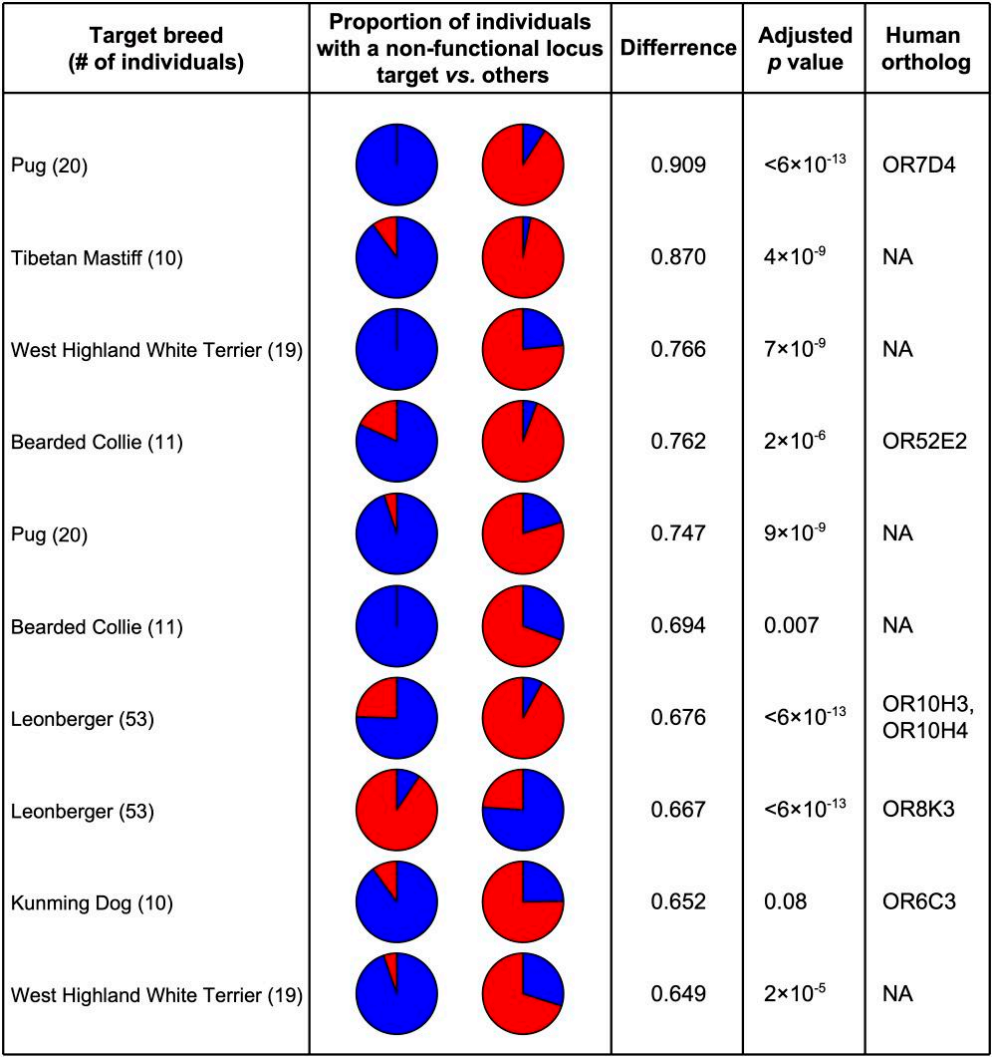
Comparison of the proportion of individuals carrying a non-functional locus between a target breed and all other breeds. We evaluated all pairwise combinations of 179 segregating pseudogenes (MAF > 1%) and each of the 14 breeds with ≥10 samples. For each combination, we calculated the difference in the proportion of individuals carrying a non-functional locus between the target breed and the remaining breeds. The top 10 comparisons with the largest differences in proportion are shown in this figure. Results for the top 50 comparisons are provided in Supplementary Table S8. Each pair of pie charts shows the proportions for the target breed (left) and all other breeds (right). In each pie chart, red indicates the number of individuals with a functional locus at the given segregating pseudogene, while blue indicates those with a non-functional locus. The name of the segregating pseudogene for each comparison is listed in Supplementary Table S8. When human orthologs of the segregating pseudogene are present, their names are shown in the “Human Ortholog” column (“NA” indicates the absence of human orthologs). For each comparison, the *P*-value from Fisher's exact test is shown after Bonferroni correction for multiple testing (14 × 179 = 2,506 comparisons).

**Fig. 6.**
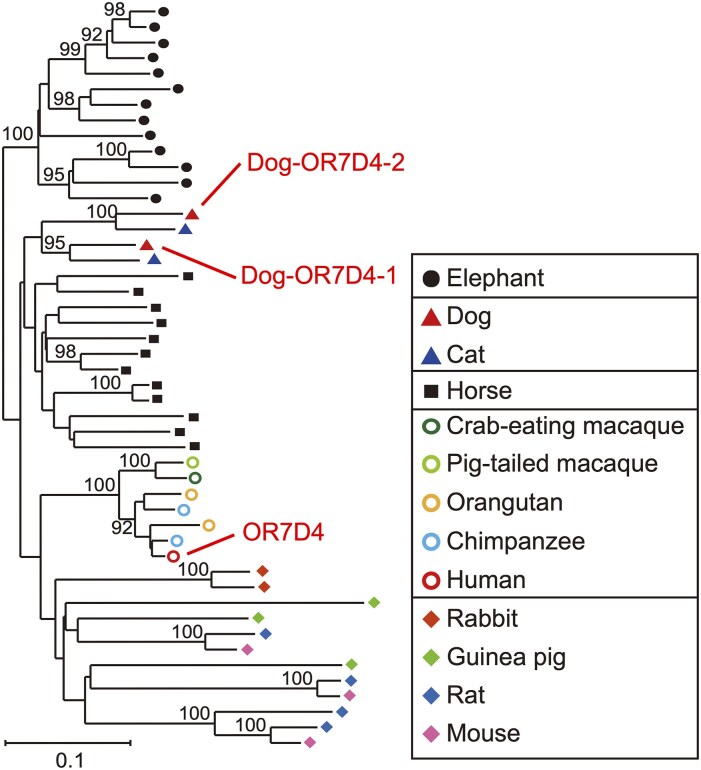
NJ phylogenetic tree of human OR7D4 orthologs. Functional OR genes from 13 mammalian species were used in the analysis. OR gene sequences for species other than dogs were obtained from (Niimura et al. 2014, 2018; Matsumoto et al. 2025). Each colored symbol represents a different species. The human OR7D4 gene is shown as a red circle, and its 2 dog orthologs (Dog-OR7D4-1 and Dog-OR7D4-2) are represented by red triangles. Bootstrap values ≥90% are shown at the corresponding nodes. The scale bar indicates the number of amino acid substitutions per site.

Finally, we searched for pseudogenization events specific to individual breeds. We calculated the difference in the proportion of individuals with a non-functional locus at each of the 179 segregating pseudogenes (MAF > 1%) between each breed (with ≥10 samples) and all other breeds (Fig. 5, Supplementary Table S7). One OR gene locus, chr20_51458187_51459125−, was found to be non-functional in all Pugs but functional in 90.9% of individuals from other breeds. This was the most extreme difference observed (*P* < 6 × 10^−13^, Fisher's exact test after Bonferroni correction). This gene is orthologous to the human OR7D4, a receptor for the pig pheromone androstenone (5α-androst-16-en-3-one), known for its variable perception among humans (Keller et al. 2007) (Fig. 6; see Discussion). In dogs, there is a second ortholog to human OR7D4, located at chr20_51273954_51274889+. We refer to these loci as Dog-OR7D4-1 (chr20_51458187_51459125−) and Dog-OR7D4-2 (chr20_51273954_51274889+), respectively. Among 15 examined dog and wolf genome assemblies, 13 (86.7%) retained a functional Dog-OR7D4-1 gene (CNV group 2-30.26 in Supplementary Table S4).

## 4. Discussion

In this study, we investigated genes encoding chemosensory receptors—ORs, V1Rs, and T2Rs—in various dog breeds and wolves. Our analysis of pseudogenizing SNVs mapped to the CanFam3.1 reference genome revealed that the number of functional OR gene loci varies considerably among dogs (Fig. 2a), as previously observed in humans (Olender et al. 2012; Mainland et al. 2014; Akhtar et al. 2022). This variation may be attributed to the combinatorial coding scheme of olfaction (Malnic et al. 1999; Saito et al. 2009), in which the relationship between odorants and receptors is not one-to-one but multiple-to-multiple; thus, a single odorant can activate multiple ORs. As a result, the pseudogenization of a few OR genes may not significantly impact an individual's fitness.

In contrast, the numbers of functional V1R and T2R genes are much more conserved: most dog individuals possess 8 and 16 functional V1R and T2R gene loci, respectively. Notably, 99.7% of individuals carry exactly 8 functional V1R loci. Although dogs retain a functional VNO, it is relatively small and structurally simpler than those of other mammals, such as mice and rats (Takami 2002; Grus et al. 2005), and is thought to have undergone retrogression (Barrios et al. 2014; Coli et al. 2016; McGlone et al. 2022). Consistent with this, dogs have a limited number of V1R genes and completely pseudogenized V2R genes. Nevertheless, the 8 V1R genes are consistently retained across nearly all individuals examined, suggesting that they may play a critical role in dogs, although their ligands and physiological functions remain unknown.

Dogs were domesticated from gray wolves approximately 15,000 yr ago. Bird et al. (Bird et al. 2021) reported that the relative size of the CP, which is closely correlated with the number of OR genes in a species' genome (Bird et al. 2018), is reduced in domestic dogs compared to wolves and coyotes. A neuroanatomical study further demonstrated that the main olfactory bulb is more developed in wolves than in dogs (Ortiz-Leal et al. 2022). These observations suggest that domestic dogs have lost some olfactory capacity during the process of domestication, possibly due to their reliance on food and shelter provided by humans. Supporting this, Mouton et al. (Mouton et al. 2025) recently reported that the estimated number of functional OR genes and the relative CP size are significantly higher in wolves than in domestic dogs.

In our SNV analyses, we did not detect significant differences in the numbers of OR, V1R, and T2R genes between dogs and wolves (Fig. 3). However, it is important to note that these SNVs were mapped to the dog reference genome, CanFam3.1, which limits our ability to detect wolf-specific genes that are absent from the dog genome. To address this limitation, we examined 2 wolf genomes that were independently assembled and not based on CanFam3.1. As a result, we found that the mCanLor1.2 assembly contains a substantially higher number of functional OR genes (861; Table 1 and Supplementary Fig. S5). Given that the number of wolves included in this study is small—8 individuals in the SNV analyses and 2 de novo assembled genomes—additional wolf genome data will be necessary for more robust and accurate comparisons.

When we compared the ratios of nonsynonymous to synonymous SNVs (N/S ratios) between dogs and wolves, we detected significant differences (Fig. 3). For both OR and T2R genes, dogs exhibited significantly higher N/S ratios than wolves. This finding suggests that functional constraints on these gene families are more relaxed in dogs, possibly as a consequence of domestication and their reliance on humans for food acquisition, as discussed above.

In the comparison among dog breed groups, we found that scent hounds did not exhibit a markedly higher number of functional OR gene loci than other breeds, despite the common belief that they have been selectively bred for enhanced olfactory abilities. This finding is consistent with previous reports indicating no significant differences between scent hounds and other breeds in either the relative CP size or the estimated number of OR genes (Bird et al. 2021; Mouton et al. 2025). However, Polgár et al. (Polgár et al. 2016) reported that scent hounds outperformed other breeds in scent detection tests. Therefore, “scent detection dogs might be chosen not so much for olfactory prowess but specifically for behavioral traits that make them more trainable,” as Bird et al. (Bird et al. 2021) suggested.

We found that Pugs had a significantly smaller number of functional OR gene loci than other dog breeds examined, and this difference became even more pronounced when we considered the number of unique functional OR genes, thereby eliminating the effect of CNVs (Fig. 4). Moreover, Pugs exhibited a significantly higher N/S ratio than other breeds. Therefore, not only has the functional OR gene repertoire been reduced in Pugs but also the functional constraint on apparently intact OR genes appears to have weakened, suggesting relaxed purifying selection in this breed.

We also detected significant differences among skull-shape types (Supplementary Fig. S8). Both brachycephalic and dolichocephalic breeds had significantly fewer unique functional OR genes and significantly higher N/S ratios than mesocephalic breeds. This observation contrasts with the findings of Mouton et al. (2025), who reported no significant differences in the estimated number of OR genes among the 3 skull-shape types. The discrepancy is likely attributable to differences in sampling: in their dataset, only 3 individuals were brachycephalic and 21 were dolichocephalic among the 111 dogs analyzed. Notably, their dataset did not include any Pugs.

Our results suggest that the functional OR gene repertoire has undergone degeneration in the Pug lineage, consistent with the widely held view that brachycephalic dogs have reduced olfactory abilities. Notably, brachycephalic breeds—particularly Pugs—are known to be prone to brachycephalic obstructive airway syndrome, a condition in which soft tissue obstructs the airway during respiration (Packer et al. 2015). A computational fluid dynamics simulation demonstrated that canine nasal airflow is optimized for odorant delivery to the MOE (Craven et al. 2010). Therefore, Pugs may experience diminished olfactory capacity due to brachycephaly, and as a result, selective pressure on OR genes may have been reduced. Alternatively, the high number of pseudogenized OR gene loci in Pugs might reflect a Pug-specific population bottleneck (Marsden et al. 2016).

We also found that an OR gene locus, Dog-OR7D4-1 (chr20_51458187_51459125−), is completely pseudogenized in Pugs, whereas it remains mostly functional (>90%) in all other breeds. This gene is orthologous to human OR7D4, which encodes a receptor for androstenone, a steroidal pheromone found in boar saliva that induces lordosis—the posture for sexual receptivity—in females. Androstenone was the first mammalian pheromone to be identified (Patterson 1968).

Perception of androstenone varies considerably among human individuals: it may be perceived as offensive (sweaty or urinous), pleasant (sweet or floral), or entirely odorless, depending on the person. Keller et al. (2007) demonstrated that these differences in perception are associated with SNVs in OR7D4. Variants of this locus include 2 non-synonymous SNVs linked to each other, R88W and T133M. Individuals with RT/WM or WM/WM genotypes were less sensitive to androstenone and rated it as less unpleasant than those with the RT/RT genotype. Furthermore, the RT-type OR7D4 receptor is activated by androstenone in vitro, whereas the WM-type receptor shows no response.

Interestingly, although androstenone is a pheromone specific to pigs, it also affects dogs: it has been shown to reduce barking and leash pulling (McGlone et al. 2014; Pirner and McGlone 2016). Given that androstenone exerts effects across species, McGlone proposed the term “interomone” to describe such cross-species semiochemicals (McGlone et al. 2022). In pigs, a receptor orthologous to human OR7D4 has recently been confirmed to respond to androstenone (Yang et al. 2025). However, it remains unclear whether Dog-OR7D4-1 is activated by androstenone, or whether there is variation in androstenone perception among dog breeds. Further studies are warranted.

In this study, we focused solely on Pugs as a brachycephalic breed, as it was the only one with more than 10 samples in the database. It remains to be determined whether the features observed in Pugs are shared by other brachycephalic breeds (e.g. Bulldogs, French Bulldogs, Boston Terriers). Moreover, it would be of interest to compare the patterns observed for chemosensory receptor genes with those of other multigene families. For example, the pancreatic amylase gene *AMY2B*, which plays a key role in starch digestion, shows increased copy number in dogs relative to wolves, likely reflecting dietary adaptation associated with the rise of agrarian societies (Axelsson et al. 2013; Arendt et al. 2016). Further research will be needed to address these questions.

In summary, we examined SNVs in OR, V1R, and T2R genes and found substantial diversity in OR gene repertoires among individual dogs, whereas the numbers of V1R and T2R genes were nearly identical across individuals. We also found evidence that functional constraints on OR and T2R genes are relaxed in dogs compared to wolves, possibly as a consequence of domestication. Furthermore, our analyses revealed that Pugs—a brachycephalic breed—possess a degenerated OR gene repertoire, consistent with the widely held view that brachycephalic dogs have reduced olfactory capabilities. Overall, this study provides valuable insights into the genetic underpinnings of chemosensory diversity among dog breeds.

## Supplementary Material

bjaf062_Supplementary_Data

## Data Availability

All data are incorporated into the article and its online supplementary material with the exception of a small proportion of the DBVDC variant information (58 of the 648 genomes) which was not previously published. These data are available on reasonable request to the DBVDC.
