# Supplementary material for: Diversity in chemosensory receptor genes in dogs and wolves: degeneration of the olfactory receptor gene repertoire in the brachycephalic Pug
Source: Chem Senses. 2025 Dec 15;50:bjaf062. doi: 10.1093/chemse/bjaf062 (PMC12703715; doi:10.1093/chemse/bjaf062)
Supplement: bjaf062_Supplementary_Data [file bjaf062_supplementary_data.zip › Supple_Figures_R1.pdf]

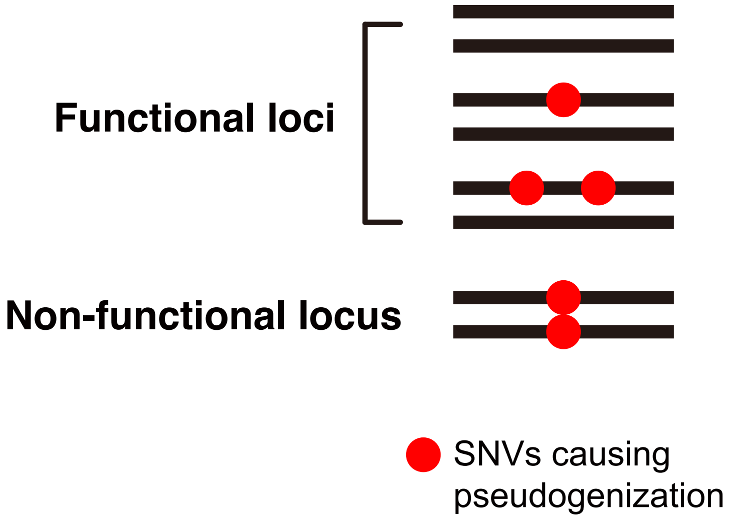

Fig. S1. Definition of functional loci. A gene locus was regarded as functional if at least one allele encoded a functional gene. When two pseudogenizing SNVs were detected within a gene, both mutations were assumed to occur on the same allele rather than on separate alleles. A locus was considered non-functional only when both alleles were pseudogenized.

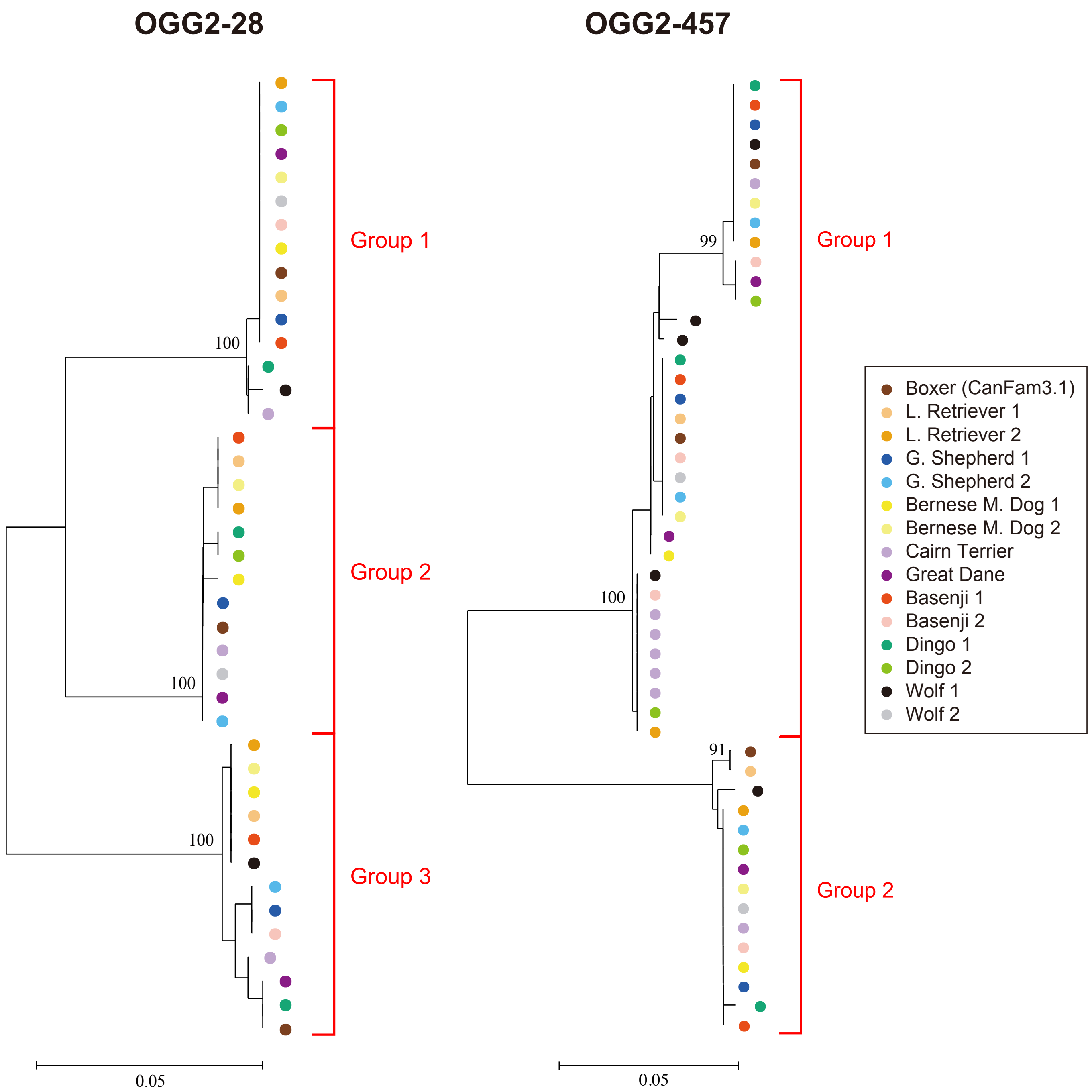

Fig. S2. Identification of CNV groups for OR genes. Phylogenetic trees for OGG2-28 (left) and OGG2-457 (right) (Niimura et al., 2014; 2018) were shown, and three and two CNV groups were identified for OGG2-28 and OGG2-457, respectively. Each OR gene is shown by a colored circle according to the right panel. Bootstrap values greater than 90% were shown. A scale bar below a phylogenetic tree indicates the number of amino acid substitutions per site.

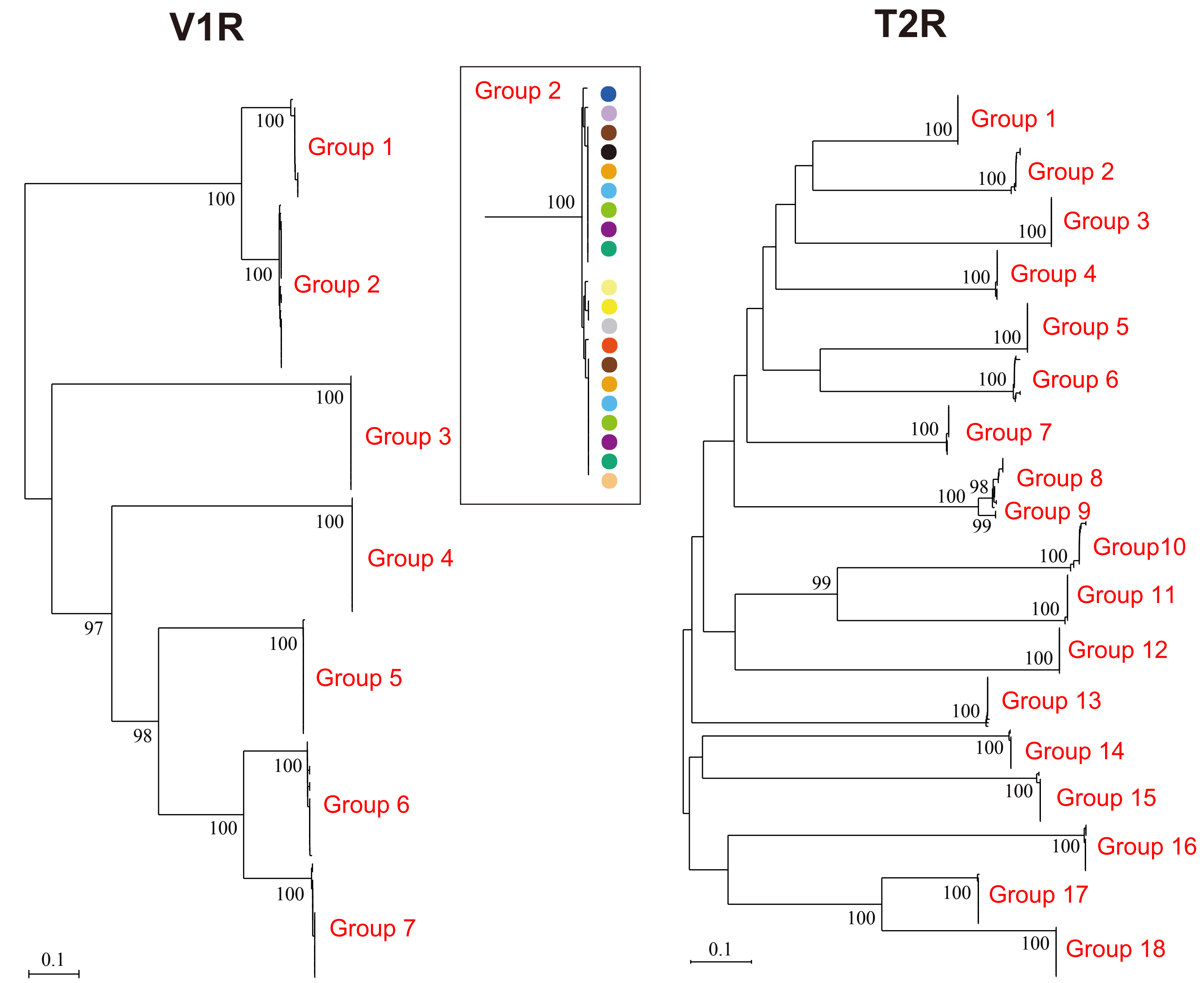

Fig. S3. Identification of CNV groups for V1R (left) and T2R genes (right). Seven and 18 CNV groups were identified for V1R and T2R genes, respectively. The inset depicts Group 2 of the V1R genes, with a color-coded circle indicating the genome assembly from which each V1R gene was identified (the color scheme is the same as in Fig. S2). This CNV group contains two genes in some genome assemblies. Bootstrap values greater than 90% are shown. A scale bar indicates the number of amino acid substitutions per site.

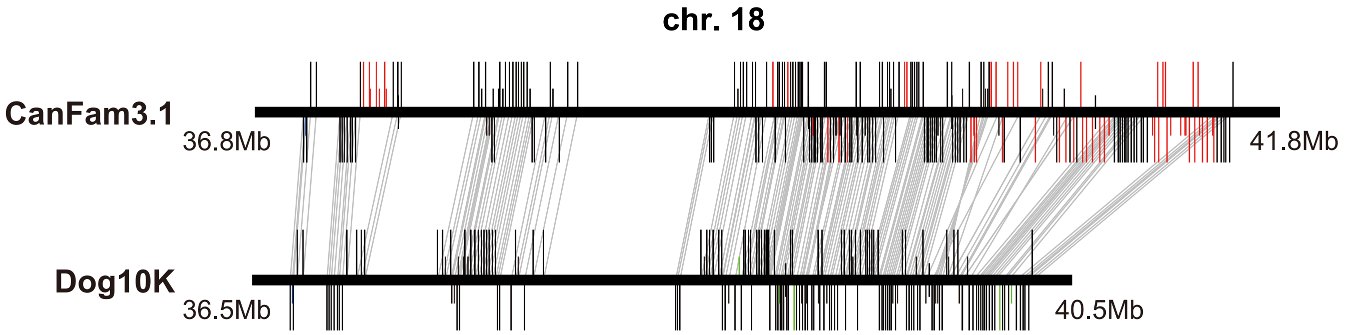

Fig. S4. Comparison of OR genes between CanFam3.1 and Dog10K assemblies. A horizontal line represents chromosome 18 in each assembly. Long and short vertical bars indicate functional OR genes and OR pseudogenes, respectively. OR genes sharing >90% amino acid sequence identity between the two assemblies are connected by gray lines. OR genes present in CanFam3.1 but absent from Dog10K are shown in red, whereas those present in Dog10K but absent from CanFam3.1 are shown in green.

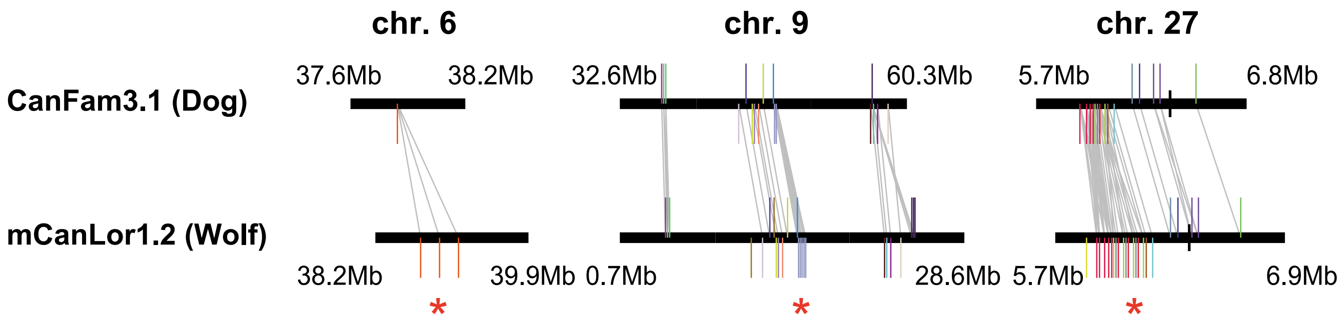

Fig. S5. Comparison of OR genes between CanFam3.1 (dog) and mCanLor1.2 (wolf) assemblies. Horizontal lines represents chromosomes 6, 9, and 27 in each assembly. Long and short vertical bars indicate functional OR genes and OR pseudogenes, respectively.Vertical bars with the same color indicate genes belonging to the same CNV group, which are connected by gray lines. An asterisk (*) indicates an increase in the number of genes within a CNV group in the wolf genome.

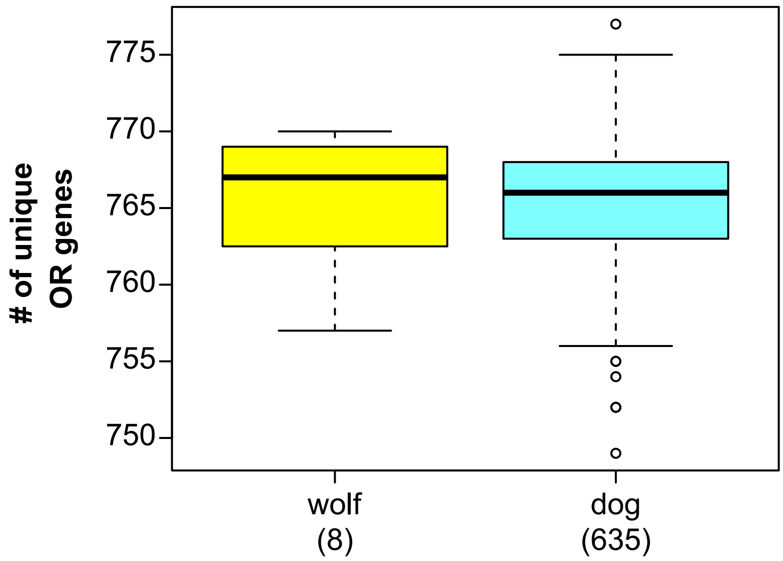

Fig. S6. Comparison of the numbers of unique OR genes between wolves and dogs.

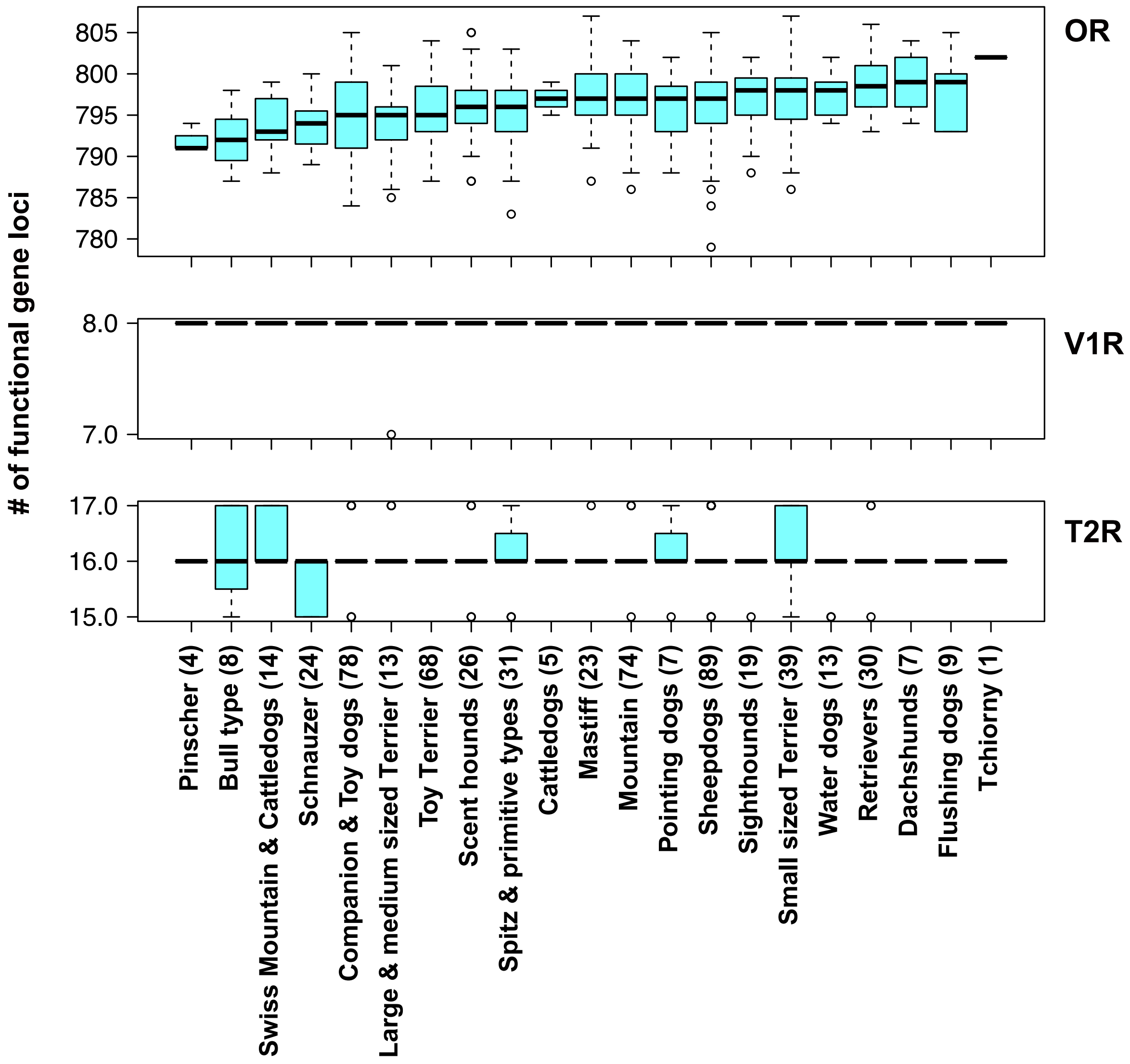

Fig. S7. Numbers of functional OR, V1R, and T2R gene loci in each breed group. The 121 dog breeds in the DBVDC database were classified into 21 FCI breed groups. Numbers in parentheses indicate the number of individuals in each group.

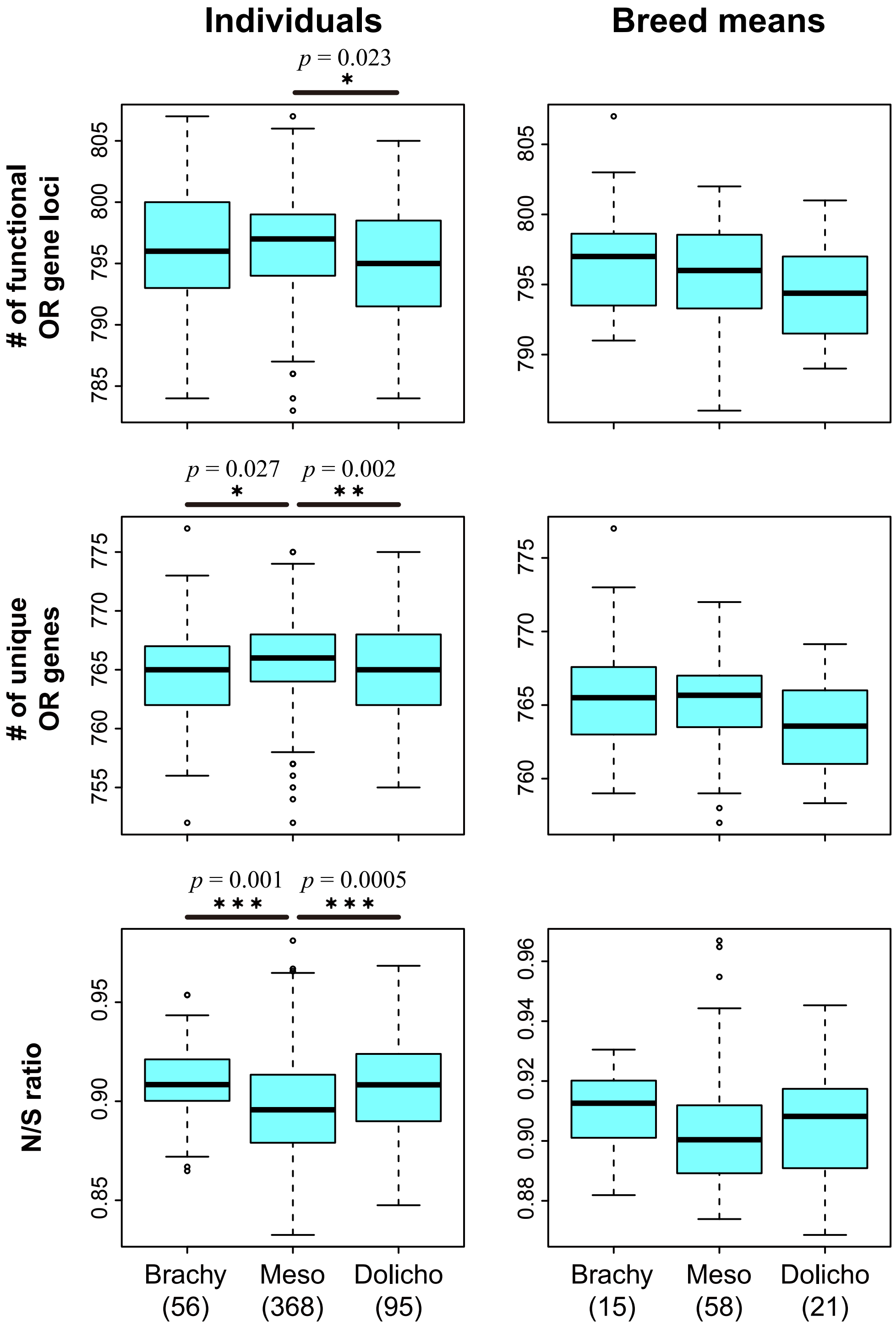

Fig. S8. Comparison of the numbers of functional OR gene loci, unique OR genes, and N/S ratios among skull-shape types based on individual values (left) and breed means (right). “Brachy”, “Meso”, and “Dolicho” denote brachycephalic, mesocephalic, and dolichocephalic breeds, respectively. Numbers in parentheses indicate the number of samples in each category. Adjusted p-values for significant pairwise comparisons obtained by Tukey’s HSD tests are also shown. ***$p < 0.001$; **$p < 0.01$; *$p < 0.05$.

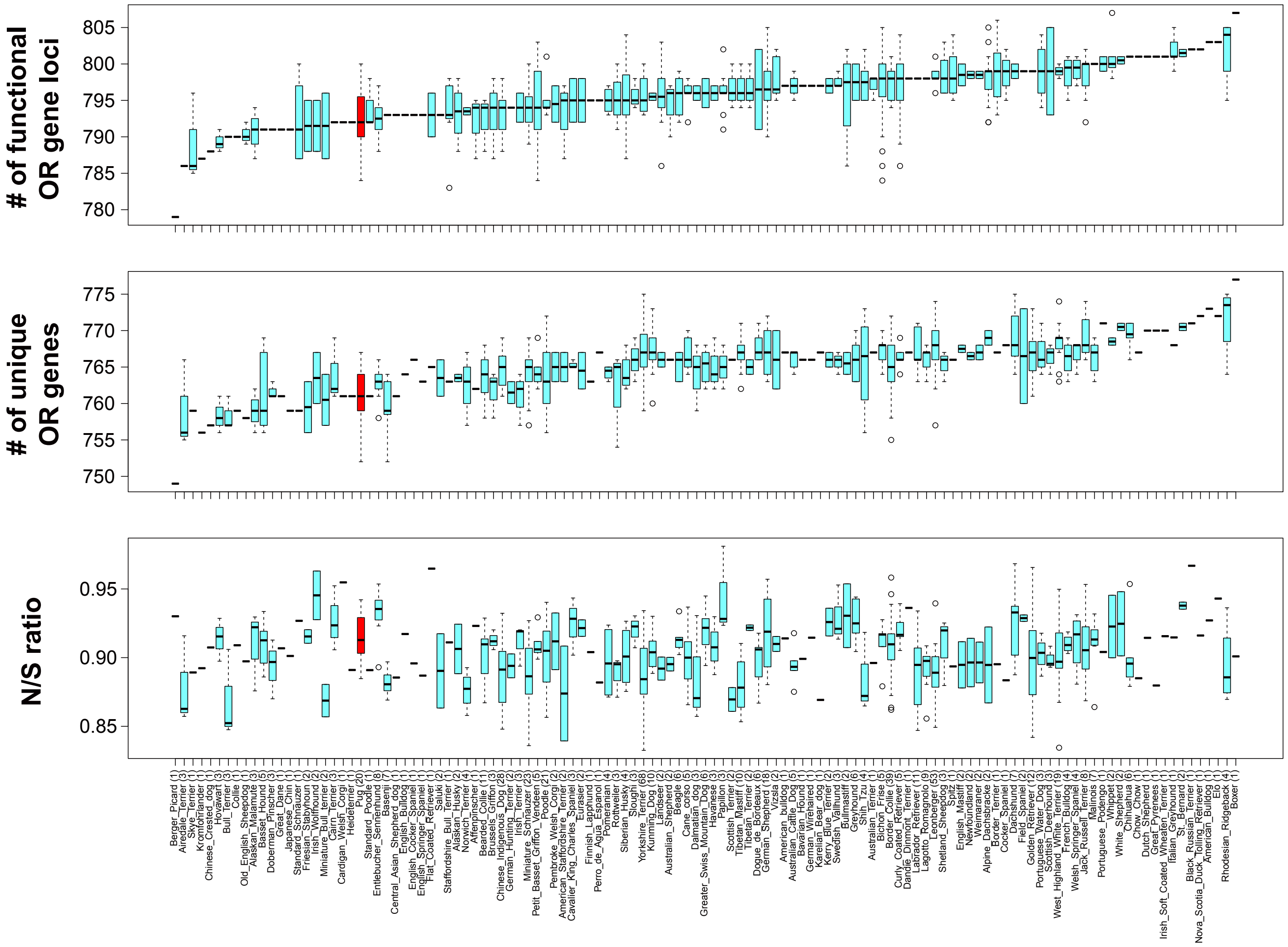

Fig. S9. Numbers of functional OR gene loci, unique OR genes, and N/S ratios among 121 dog breeds. Numbers in parentheses indicate the number of individuals in each breed. Pug data are shown in red.

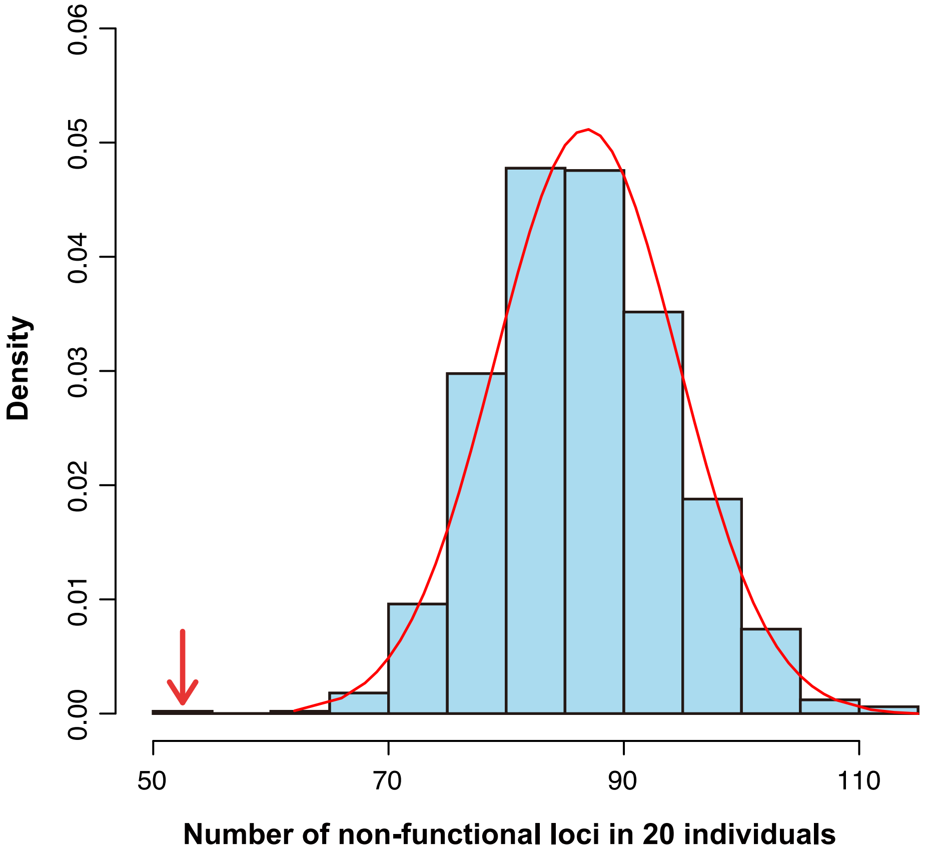

Fig. S10. Distribution of the number of pseudogenized loci present in at least one individual among 20 randomly selected dogs. The red line represents a normal distribution fitted to the simulated data (mean = 86.3, standard deviation = 7.79). The red arrow indicates the corresponding value for 20 Pugs (54).
